# Nitrate of Silver in Whitlow

**Published:** 1886-11-20

**Authors:** 


**Nitrate of Silver in Whitlow.**—I was once called upon to treat two cases of whitlow in the same family. They were as near alike as possible. One I treated with poultices, etc., and in the other case I painted the whole surface of the first joint of the finger with the solid stick of nitrate of silver. A hard crust was formed, which had to be removed to give exit to the pus. Both suppurated, and discharged; but the pain was far less in the one to which I had applied the silver. The latter was well in three weeks; the other required over six weeks.— *W. in Medical World.*

